# Electricity Load Forecasting Using Support Vector Regression with Memetic Algorithms

**DOI:** 10.1155/2013/292575

**Published:** 2013-12-26

**Authors:** Zhongyi Hu, Yukun Bao, Tao Xiong

**Affiliations:** Department of Management Science and Information Systems, School of Management, Huazhong University of Science and Technology, Wuhan 430074, China

## Abstract

Electricity load forecasting is an important issue that is widely explored and examined in power systems operation literature and commercial transactions in electricity markets literature as well. Among the existing forecasting models, support vector regression (SVR) has gained much attention. Considering the performance of SVR highly depends on its parameters; this study proposed a firefly algorithm (FA) based memetic algorithm (FA-MA) to appropriately determine the parameters of SVR forecasting model. In the proposed FA-MA algorithm, the FA algorithm is applied to explore the solution space, and the pattern search is used to conduct individual learning and thus enhance the exploitation of FA. Experimental results confirm that the proposed FA-MA based SVR model can not only yield more accurate forecasting results than the other four evolutionary algorithms based SVR models and three well-known forecasting models but also outperform the hybrid algorithms in the related existing literature.

## 1. Introduction

Electricity load forecasting has always been the essential part of efficient power system planning and operation. Specially that it is not only critical for automatic generation control, reliable operation, and resource dispatch but is also a fundamental piece of information used for energy transactions in competitive electricity markets [1]. Inaccurate forecast of power load leads to a great deal of loss for power companies, and a 1% increase in forecasting error implied a 10 million increase in operating costs [2]. However, the electricity load is inevitably affected by various factors such as climate factors, social activities, and seasonal factors, thus making it difficult to be accurately predicted.

During the past several decades, numerous approaches have been proposed for electricity load forecasting. Traditional methods, such as autoregressive moving average model (ARMA) [3], exponential smoothing models [4, 5], and regression models [6, 7] are often difficult to model the electricity load with high accuracies due to the nonlinearity of the load inherently. On the other hand, with the development of intelligence techniques in recent years, many studies have tried to apply the artificial intelligence techniques to improve the forecasting accuracy of load. Among them, neural networks (NN) have received much share of attention, and a great number of studies have reported successful results in the load forecasting [8]. Refrence [9] proposed a practical method using NN combined similar days approach, which resulted in a reliable forecasts for one-to-six hour-ahead electricity load. Refrence [10] proposed an adaptive artificial neural network with particle swarm optimization (PSO) used to adjust the network's weights; computational results indicated that the proposed model can obtain higher forecasting precision with traditional BP algorithm. Refrence [11] applied Bayesian neural network in the short-term load forecasting and the results of the proposed model gains better performance than that of conventional neural networks. Neural network with novel learning algorithm based on a modified harmony search technique also gives better results than several benchmarks [12]. Refrence [8] gave a comprehensive review and evaluation of the neural networks for short-term load forecasting. However, the NN has a large number of parameters to be tuned and suffers from the danger of over-fitting.

Different from NN which minimizes the empirical error based on the empirical risk minimization principle (ERM), support vector regression (SVR) implements the structural risk minimization principle (SRM) by minimizing an upper bound to the generalization error [13]. This leads to excellent generalization performance with SVR, which has been shown to outperform other nonliner forecasting techniques including NN based forecasting models [14]. As one of the fields in time series forecasting using SVR [14–20], electricity load forecasting using SVR as well as its varieties has been well studied. Refrence [16] proposed a locally weighted support vector regression to solve the short-term load forecasting problem, and the experimental results proved the superior performance of the proposed model compared with some published models. Combined with fuzzy c-means (FCM) and particle swarm optimization (PSO), a SVR based model is studied to forecast the short-term load of a city [21]. Refrence [17] proposed a TF-*ε*-SVR model with trend fixed and seasonal adjustment to improve the forecasting accuracy of the electricity demand.

However, the generalization ability of SVMs highly depends on the adequate setting of parameters [22–25], such as penalty coefficient, kernel parameters, and the width of loss function. Therefore, the selection of the optimal parameters is of critical importance to obtain a good performance in handling electricity load forecasting task with SVR. Recently, various studies try to improve the forecasting accuracy of electricity load when the SVR model is used. Refrence [18] employs simulated annealing algorithms (SA) to choose the parameters of SVR, and computational results show that the SA based SVR model achieves better performance for load forecasting compared with autoregressive integrated moving average (ARIMA) model and the general regression neural networks (GRNN) model. Refrence [26] proposed a differential evolution algorithm based SVR model to forecast the annual load. Refrence [27] proposed a LSSVM based load forecasting model with the fruit fly algorithm used to automatically choose the parameters of LSSVM; experimental results show that the proposed model outperforms some other alternative models. Pai et al. conducted a series of relevant researches by using genetic algorithm (GA) [28, 29], chaotic particle swarm optimization (CPSO) [30], artificial bee colony algorithm (ABC) [31], immune algorithm (IA) [32, 33], and hybrid algorithm [34] for parameters determination of SVR to improve the forecasting accuracy of the electricity load. However, GA and some other evolutionary algorithms (EAs) are not guaranteed to find the global optimum parameters of a SVR model, though they are generally good at finding “acceptable good” or near-optimal solutions to problems. More specifically, although they are good at exploring the solution space and detecting the region of attraction of the global optimum efficiently, they lack the abilities to perform a refined tuning search locally [24, 35].

Memetic algorithms (MAs), a powerful algorithmic paradigm that combines the evolutionary algorithms (EAs) with problem-specific local searcher (LS), have been successfully applied in a wide variety of areas [36–38]. MA has the ability to exploit the complementary advantages of EAs (generality, robustness, and global search efficiency), and problem-specific local search (exploiting application-specific problem structure, rapid convergence toward local minima) [39]. In our previous study [24], MA is proposed to tune the parameters of SVM in classification problems. However, there are, if any, few works related to Mas that have been reported in the load forecasting literature on the issue of SVR parameters optimization. Notice that there are three important parameters in SVR, whereas SVM for classification has only two. With the increase of dimensions and the change of structure complexity for the optimization problem, the performance of MA is a big challenge. As such, it is of interest to involve the MAs for SVR parameters optimization in improving the prediction accuracy of STLF. In this study, by combining firefly algorithm (FA) and pattern search (PS), an efficient FA based memetic algorithm (FA-MA) is proposed to automatically determine the parameters of SVR for improving the forecasting accuracy of electricity load forecasting. In the proposed FA-MA, FA is responsible for the exploration of the search space and the detection of the potential regions with optimum solution, while PS is used to produce an effective exploitation on the potential regions obtained by FA. The performance of proposed FA-MA for parameters optimization in SVR is justified on two real-world cases against selected counterparts.

The rest of the study is organized as follows.  Section 2 presents a brief review on SVMs.  Section 3 elaborates on the FA-MA proposed in this study. The results with discussions are reported in Section 4. Finally, we conclude this study in Section 5.

## 2. Support Vector Regression

Given a set of training data, {(*x*_1_, *y*_1_), (*x*_2_, *y*_2_),…, (*x*_*n*_, *y*_*n*_)} ⊂ *R* ^*n*^ × *R*, where *x*_*i*_ is input vector, *y*_*i*_ is the target, and *n* denotes the number of the data items in the training set. Based on the structured risk minimization (SRM) principle [13], rather than finding minimum empirical errors, SVMs aim to generate a decision function (1) by minimizing a regularized risk function (2)

(1) $$\begin{array}{c} f\left(x\right)=\langle w,\phi \left(x\right)\rangle +b, \end{array}$$

(2) R=Remp+12||w||2    =Cn∑i=1nL(yi,f(x))+12||w||2,

(3) $$\begin{array}{c} L\left(y_{i},f\left(x\right)\right)=\{\begin{array}{cc} \left|y-f\left(x\right)\right|-\varepsilon & \text{if}\;\;\left|y-f\left(x\right)\right|\geq \varepsilon \\ 0 & \text{otherwise}, \end{array} \end{array}$$

where in (1), 〈,〉 denotes the inner product, *w* is the weight vector, that controls the smoothness of the model, and *b* is a parameter of bias. *ϕ*(*x*) is the high-dimensional feature space which is nonlinearly mapped from the input space *x*. In the regularized risk function given by (2), the first term *R*_emp_ or (*C*/*n*)∑_*i*=1_^*n*^ *L*(*y*_*i*_, *f*(*x*)) is the empirical risk. In SVR, Vapnik's *ε*-insensitive loss function [40] given by (3) is often used to measure the empirical risk, and *ε* is called the tube size. The second term, (1/2)||*w*||^2^, is the regularization term to be used as a measure of flatness or complexity of the function. Hence, *C* is referred to as the regularized constant and it specifies the trade off between the empirical risk and the regularization term. Both *C* and *ε* are user-determined parameters.

By introducing two positive slack variables *ξ* and ξ*, (2) is transformed into the following constrained form:

(4) Minimize   R=C∑i=1n(ξi+ξi∗)+12||w||2Subjected  to    yi−〈w,xi〉−b≤ε+ξi          〈w,xi〉+b−yi≤ε+ξi∗           ξi,ξi∗≥0,  i=1,2,…,n.

According to Wolfe's dual theorem and the saddle-point condition, the dual optimization problem of the above primal one is obtained as in the following form:

(5)   max⁡α,α∗ −12∑i,j=1l(αi−αi∗)(αj−αj∗)〈ϕ(xi),ϕ(xj)〉−ε∑i,j=1l(αi+αi∗)+∑i,j=1lyi(αi−αi∗)  s.t.  ∑i,j=1l(αi−αi∗)=0, αi,αi∗∈[0,C]

with

(6) $$\begin{array}{c} w=\sum_{i=1}^{n}\left(\alpha_{i}-\alpha_{i}^{*}\right)\phi \left(x_{i}\right), \end{array}$$

where *α*_*i*_, *α*_i_* are nonnegative Lagrange multipliers that can be obtained by solving the convex quadratic programming problem stated above.

Finally, based on the (6) and the trick of kernel function, the decision function given by (1) has the following explicit form:

(7) $$\begin{array}{c} f\left(x\right)=\sum_{i=1}^{n}\left(\alpha_{i}-\alpha_{i}^{*}\right)K\left(x_{i},x_{j}\right)+b. \end{array}$$

Here, *K*(*x*_*i*_, *x*_*j*_) is defined as kernel function. The value of the kernel function is equivalent to the inner product of two vectors *x*_*i*_ and *x*_*j*_ in the feature space *ϕ*(*x*_*i*_) and *ϕ*(*x*_*j*_); that is, *K*(*x*_*i*_, *x*_*j*_) = 〈*ϕ*(*x*_*i*_), *ϕ*(*x*_*j*_)〉. The elegance of using the kernel function is that one can deal with feature spaces of arbitrary dimensionality without having to compute the map *ϕ*(*x*) explicitly. Any function that satisfies Mercer's condition [40] can be used as the kernel function. There are several typical examples of kernel function such as linear kernel, polynomial kernel, radial basis function (RBF), and sigmoid kernel. Each kernel has some parameters. Generally, among these kernel functions, RBF kernel (8) is strongly recommended and widely used for its performance and complexity [41] and thus SVR with RBF kernel function is the one studied in this study.

Consider

(8) $$\begin{array}{c} K\left(x_{i},x_{j}\right)=\exp\left(\frac{-{||x_{i}-x_{j}||}^{2}}{2\delta^{2}}\right), \end{array}$$

where *δ* is kernel parameter. The kernel parameter should be carefully chosen as it implicitly defines the structure of the high-dimensional feature space *ϕ*(*x*) and thus controls the complexity of the model.

Overall, SVR is a powerful learning machine with strong theoretical foundations and excellent generalization performance. Note that before implementing the SVR with RBF kernel, there are three parameters (penalty parameter *C*, RBF kernel parameter *δ*, and width of *ε* loss function) to be set. Previous studies show that these three parameters play an important role in the success of SVR [42]. In this study, to determine these parameters and to improve the forecasting accuracy of SVR in electricity load forecasting, a firefly algorithm (FA) based memetic algorithm (FA-MA) is proposed in Section 3.

## 3. Memetic Algorithm for Parameters Selection of SVR

Memetic algorithms (MAs), one of the recent growing areas in computational intelligence, is first coined by Moscato and Norman [43]. Inspired by Darwinian principles of natural evolution and Dawkins' notion of meme, MA has come to light as an union of population based stochastic global evolutionary algorithm and local improvement procedures. As a designed a hybridization, MAs are expected to make full use of the balance between exploration and exploitation of the search space to complement the advantages of population based methods and local based methods. Nowadays, MAs have revealed their successes with high performance and superior robustness across a wide range of problem domains; detail reviews are reported in [44, 45]. Since often there are no free lunches, the hybridization can be more complex and expensive to implement. Considering the effectiveness of firefly algorithm which is introduced recently and can be even superior to the GA and PSO [46–48], this study proposed a FA based memetic algorithm with pattern search as a local individual learner, to improve the forecasting accuracy of electricity load forecasting model using SVR.

In the following subsections, we will explain the implementation of the proposed FA-MA for parameters optimization in details.

### 3.1. Initialization

In the proposed FA-MA, each firefly (or individual) is a parameter set of the SVR model and can be denoted as **x**_*i*_ = 〈*C*, *δ*, *ε*〉. A set of fireflies is called a swarm or population. Traditionally, initial swarm is often generated randomly in firefly algorithm or other evolutionary algorithms. To guarantee an initial swarm with reliability and diversity, Latin hypercube sampling (LHS) method is applied to generate a random sample set. With the use of LHS, we first split the search space into subspaces and then try to take randomly the values within each subspace to achieve an initial sample set which is representative of the whole search space. Hence, it can guarantee the initial samples to be relatively uniformly distributed over each dimension, which is proved to be superior to random initialization [49].

### 3.2. Fitness Function

Since the ultimate goal of the SVR model is to forecast the future electricity load with high accuracy (i.e., known as generalization ability), it is important to choose such fitness function which can estimate the generalization ability when determining the parameters in SVR with FA-MA. In this study, the data is split into three parts which are training set, validation set, and testing set. The training set is used to train the SVR model with a certain parameter set, and the validation set is deserved to assess the generalization ability of the established forecasting model. The parameter set with lowest mean squared percentage error (MAPE) (For convenience, the formulation of MAPE is given in Section 4.2.) in the validation set is selected as the optimal solution. That is to say, MAPE in the validation set is used as the fitness function. In the proposed firefly algorithm based memetic algorithm, the brightness or light intensity of a firefly is determined by the fitness function.

### 3.3. Exploration with Firefly Algorithms (FAs)

Firefly algorithm, first introduced by Yang et al. [46, 47], is a swarm based intelligent metaheuristic. The FA mimics the social behavior of fireflies which move and communicate with each other based on their flashing characteristics, such as brightness, frequency, and the time period. Specially, the superiority of FA against genetic algorithms (GAs) and particle swarm optimization (PSO) in existing studies [46, 47] motivates us to use the FA to explore the search space.

In FA, each firefly is assumed to be attracted to other ones regardless of their sex, and the attractiveness is proportional to their brightness. Besides, as mentioned before, the brightness of a firefly is determined by the fitness function. To minimize the fitness defined in Section 3.2, the brightness can simply be minus of the MAPE.

The movement of a firefly *i* attracted by another more attractive firefly *j* can be formulated as (Details of the definition are shown in Yang [46].)

(9) $$\begin{array}{c} v_{i}=v_{i}+\beta_{0}e^{-\gamma r_{ij}^{2}}\left(v_{j}-v_{i}\right)+\alpha \left(\text{rand}-\frac{1}{2}\right), \end{array}$$

where second term is the attraction of firefly *j* to firefly *i*, and the third term is the randomization of the movement. *γ* is a absorption coefficient, *r*_*ij*_ is the Cartesian distance between two fireflies *i* and *j*. *β*_0_ is the attractiveness at *r*_*ij*_ = 0, *α* is a randomization parameter, rand is a random number generator uniformly distributed in [0,1]. As recommended by [46], *γ* = 1, *β*_0_ = 1, and *α* ∈ [0,1] are used in this study. Besides, *α* is often replaced by a *αS*_*k*_ where the scaling parameters *S*_*k*_ is determined by the actual scales of the problem.

### 3.4. Refinement with Pattern Search

In the proposed FA-MA, pattern search is employed to conduct exploitation of the parameters solution space. Pattern search (PS), a simple effective optimization technique, has already been successfully used in parameters optimization in previous studies [50]. By examining the neighborhood of the current solution, pattern search is very effective to exploit the local regions. In addition, its convergence to local minima for constrained problems as well as unconstrained problems has been proven in [51]. Thus, it is deserved to enhance the local exploitation of the FA in proposed memetic algorithm. In some sense, the main objective of PS is to conduct individual learning by exploiting small local regions effectively in relatively short periods of time.

Pattern search investigates nearest neighborhood of the current solution and tries to find a better move. If all neighbors fail to produce an improvement, then the search step is reduced. This search stops until the search step gets sufficiently small, ensuring the convergence to a local minimum. The pattern search is based on a pattern *P*_*k*_ that defines the neighborhood of current solution. A well often used pattern is five-point unit-size rood pattern which can be represented by the generating matrix *P*_*k*_ in (10):

(10) $$\begin{array}{c} P_{k}=\left[\begin{array}{cccccc} 1 & 0 & 0 & -1 & 0 & 0\\ 0 & 1 & 0 & 0 & -1 & 0\\ 0 & 0 & 1 & 0 & 0 & -1 \end{array}\right]. \end{array}$$

The procedure of pattern search is outlined in Algorithm 1. Δ_0_ denotes the default search step of PS, Δ is a search step, *p*_*k*_ is a column of *P*_*k*_, and *Ω* denotes the neighborhood of the current solution. The termination conditions are the maximum iteration is met or the search step gets a predefined small value. To balance the amount of computational budget allocated for exploration versus exploitation, Δ0/8 is experimentally selected as the minimum search step.

### 3.5. Description of the Proposed FA-MA

Based on the population initialization, firefly algorithm based exploration, and pattern search based individual learning, a FA-MA is illustrated in Algorithm 2.

It can be seen that FA-MA not only applies the FA to effectively perform exploration for promising solution in the whole search space but also employs pattern search to perform exploitation for individual learning in local spaces. To guarantee an initial swarm with diversity, Latin hypercube sampling method is applied to generate a random sample set. In addition, it is important to balance the exploration and exploitation under limited computational budget in MA. Hence, in this study, each firefly undergoes local refinement with a specified probability pl(*x*_*k*_), and the selection probability is defined by a roulette wheel section scheme with linear scaling [24]:

(11) $$\begin{array}{c} \text{pl}\left(x_{k}\right)=\frac{f_{\max}\left(P\right)-f\left(x_{k}\right)}{\sum_{y\in P}^{}\left(f_{\max}\left(P\right)-f\left(y\right)\right)}, \end{array}$$

where *f* is a fitness function (i.e., MAPE in this study) and *f*_max⁡_(**P**) is the maximum fitness value among the current population **P**. With this selection probability, a firefly with better fitness value gains more chance to be selected for exploitation.

Since both exploration and exploitation are stressed and balanced, it is expected to have good ability for improving the load forecasting with SVR. In the next section, we will investigate the performance of the proposed FA-MA.

## 4. Experimental Results

### 4.1. Experimental Setup

To verify the electricity load forecasting performance of the proposed SVR-MA model, two real-life cases are considered in this study. The first one is the hourly observations from Pennsylvania-New Jersey-Maryland (PJM) power system, which is a well-established electricity market in U.S. The data consists of 18 months of hourly observations, from January 1, 2010, to 31 June, 2011 (data are available from PJM Interconnection, http://www.pjm.com). The series consists of 13104 hourly observations. The second one is the monthly electric load of Northeast China which has been investigated in the existing literature [34]. This data consists of 64 monthly observations with the date from January 2004 to April 2009.

As a preprocessing stage, several missing load values are filled in by the average of the neighboring values. By adopting linear transformation (in (12)), the series are linearly scaled to the range [0,1]. The main advantage of scaling is to avoid attributes in greater numeric ranges dominating those in smaller numeric ranges. Another advantage is to prevent numerical difficulties during the calculation [41]

(12) $$\begin{array}{c} x^{\prime }=\frac{x-\text{mi}{\text{n}}_{A}}{\text{ma}{\text{x}}_{A}-\text{mi}{\text{n}}_{A}}, \end{array}$$

where *x* is an original value of attribute *A*, *x*′ is the scaled value, min_*A*_ is the minimum of attribute *A*, and max_*A*_ is the maximum of attribute *A*. It should be noted that the forecasting value will be rescaled back following the reverse of the linear transformation and the forecasting performance is calculated based on the original scale of the data.

### 4.2. Performance Measures

To assess the forecasting performance of a model, many accuracy measures can be used [52]. However, it is hard to say whether one accuracy measure is better or worse than the other [53]. Besides, different metrics may evaluate the quality of the forecasting performance from different perspectives. In this study, three accuracies are selected to assess the prediction performance, they are mean absolute percentage error (MAPE), mean absolute scaled error (MASE), and directional symmetry (DS). The definitions of them can be found in Table 1. MAPE is one often used metric, which measure the percentage error between the actual and predicted values. The smaller the values of MAPE, the closer the predicted values to the actual values. MASE is a scaled error which is scaled by a naïve forecast model. MASE is less than one if the forecast is better than the naïve method, and the smaller the values of MASE, the better the naïve method. It is highly recommended in recent study as it is less sensitive to outliers and easy to be interpreted [52]. DS provides an indication of the accuracy of the predicted direction and the large value suggests a better predictor.

Furthermore, a nonparametric Wilcoxon's signed-rank test [54] is performed to determine if there is significant difference between the two approaches based on the prediction error of the testing data sets. This test performs a two sample rank test for the difference between two population medians. Since the population distributions of the performance measures are unknown, a nonparametric test is suggested for the performance comparison of the two models [55].

### 4.3. Results and Discussions

In the first case, the 24 step-ahead electricity load is predicted directly. The data are divided into three parts: training set, validation set, and testing set. The periods and number of observations of each set are shown in Table 2. As mentioned in Section 3.2, the training set and validation set are used to determine the optimal parameters, and then the forecasting model is established in the integrated training set (training set and validation set). At last, the testing set is used to assess the out-of-sample forecasting performance of the proposed model with optimal parameters obtained by memetic algorithms. Considering the short-run trend, daily and weekly periodicity characteristics of hourly load, the hourly load values of the last one day, and the similar hours in the previous 30 days are selected as the input variables set of the forecasting model. Then, the input variables are selected from the variables set by a filter method which maximizes the mutual information using forward-backward selection strategy [56].

To verify the improvement of forecasting accuracy with our proposed memetic algorithm in SVR based forecasting model, four well-known evolutionary algorithms (EAs) including genetic algorithm (GA), particle swarm optimization (PSO), simulated annealing (SA), and firefly algorithm (FA) are selected to determine the parameters (*C*, *δ*, and *ε*) in SVR based load forecasting model. The experiments are implemented in MATLAB 2012a using computer with Intel Core 2 Duo CPU T5750, 2.00 GHZ, and 2 G RAM. The parameters' search space in SVR is defined as an exponentially growing space: log_2_ *C* ∈ [−6,6], log_2_ *γ* ∈ [−6,6], and log_2_ *ε* ∈ [−6,6]. The parameters in each EA are controlled based on initial experiments. More specifically, the population size of each method is set as 30, and the stopping criterions of each method are set as follows: the number of iterations reached 150 or there is no improvement in the fitness for 50 consecutive iterations. The scaling parameters *S*_*k*_ in firefly algorithm and FA-MA are both set as 1, which is 1/6 percentage of the maximum of search space.

The forecasting results of different EAs based SVR forecasting model in each separate month and the whole testing period are illustrated in Tables 3, 4, and 5. For the purpose of reducing statistical errors, the results in Tables 3–5 are average results of 30 independently trial runs. From Tables 3–5, several observations can be drawn. Firstly, compared with GA, PSO, and SA, the FA based forecasting model can obtain the best performance in most of the periods for each metric, which imply the superior ability of determining the parameters in SVR and thus improving the forecasting performance. Secondly, by using the proposed MA to determine the parameters in SVR forecasting model, the forecasting results outperform the FA based forecasting model. The superior performance against FA can be contributed to the integration of pattern search for finely exploitation and the balance between exploration and exploitation in proposed MA. Thirdly, the proposed MA has the lowest MAPE and MASE, with the largest DS, which confirms the superiority of MA in improving the forecasting accuracy by enhancing the parameters determination process of SVR forecasting model.

Table 6 reports the time consumption of each evolutionary algorithm in selecting the optimal parameters in SVR forecasting model. From Table 6, we can see that the proposed FA-MA is a little more time consuming than that of the other four methods, which is mainly due to the finely exploitation with pattern search. However, in the real-world applications, this computational time is acceptable within a day-ahead decision making framework, and considering the forecasting performance of FA-MA in improving the forecasting, it can be used as an alternative method to improve the forecasting accuracy when the support vector regression (SVR) is used.

**Figure 1:**
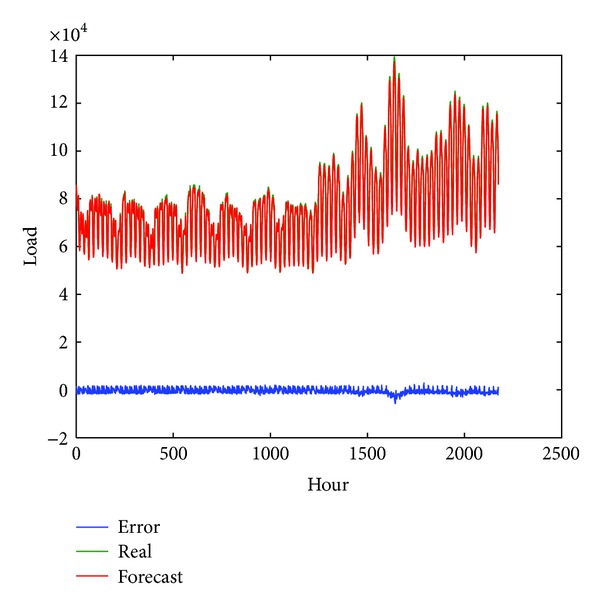
Curves of real values, forecast values, and errors of proposed FA-MA based SVR model.

Furthermore, three well-known forecasting model, including radial basis function neural network (RBFNN), MLP neural network trained by LM (Levenberg-Marquardt), and autoregression integrated moving average (ARIMA), are selected to compare the day-ahead forecasting performance of the proposed FA-MA based SVR model. For the sake of fair comparison, the above two neural network based models have the same process in data preprocess, input selection, and parameters tuning to our proposed FA-MA based SVR model. While for ARIMA, the *forecast* package [57] in *R* is used to forecast the load. Tables 7, 8, and 9 show the comparison of average results of three separated months and the whole period. From Tables 7–9, it can be observed that the ARIMA is the worse one in each month and the whole period, which is mainly due to the linearity assumption. Besides, the proposed FA-MA based SVR model outperforms all the other forecasting models in terms of MAPE, MASE, and DS. Moreover, to verify the significance of accuracy improvement of proposed FA-MA based SVR model, a nonparametric Wilcoxon's signed-rank test is used to test the significant different of FA-MA based SVR with three other models. The significant test shows that our proposed model is statistically superior to others at the 0.05 significance levels. In addition, to give a graphical view about the forecasting performance of the proposed model, the curves of real values, forecast values, and forecast errors are shown in Figure 1. It is obviouse that the forecast curve accurately predicts the real values and only minor errors are obtained. The figure further illustrates the effectiveness of our proposed forecasting model.

In addition, to further verify the proposed performance of FA-MA against the existing hybrid methods, the second case, obtained from previous studies [34], is applied here. Similar to previous studies [34], the last 7 months are predicted. The seasonal mechanism effects stated in [34] are also taken into consideration.  Table 10 shows the actual values and the forecasting load obtained by different forecasting models. The TF-*ε*-SVR-SA reports the results from [17]; the SVR model is optimized by SA. For CGASA and S-CGASA, the forecasts were generated by the SVR model with or without the seasonal mechanism, respectively. The parameters in these two SVR models were tuned by a hybrid algorithm, namely, chaotic genetic algorithm-simulated annealing algorithm (CGASA). In the last two columns, the SVR models were optimized by our proposed FA-MA algorithm. The only difference between them is that the S-FA-MA takes the seasonal mechanism effects into account. As illustrated in Table 10, the proposed FA-MA gains smaller MAPE, MASE than TF-*ε*-SVR-SA and CGASA. However, the results of SVRFA-MA is worse than those of S-CGASA, which is mainly due to an involvement of a seasonal mechanism in S-CGASA. Similar to S-CGASA, S-FA-MA also makes full use of the seasonal effects; it generates superior performance to S-CGASA in terms of MAPE, MASE and has competitive performance with S-CGASA in terms of DA. Thus, in this case, we can conclude that the proposed FA-MA is superior to the existing hybrid SA, CGASA in improving the forecasting accuracy of SVR models. The outstanding forecasting performance of our proposed FA-MA against the existing hybrid algorithm (i.e., CGASA) is caused by the following reasons. Firstly, based on the framework of memetic algorithm, both global exploration and local exploitation are enhanced in the proposed FA-MA, which not only can avoid the premature convergence but also ensure searching capability. Secondly, a roulette wheel section scheme is applied in the proposed FA-MA to select the individuals to be refined, which generates a good balance to the exploration and exploitation.

## 5. Conclusions

Electricity load forecasting is an important issue to operate the power system reliably and economically. In this study, to improve forecasting accuracy of electricity load forecasting using support vector regression (SVR), a firefly algorithm (FA) based memetic algorithm (FA-MA) was presented. In the proposed FA-MA, FA was employed to explore the search space and detect the potential regions, while pattern search (PS) was used to conduct the individual learning to improve the exploitation ability of FA. With the proposed FA-MA used to determine the parameters of SVR, a novel forecasting model, FA-MA based SVR, was presented to forecast the electricity load with two real cases. In the first case, four evolutionary algorithms (FA, GA, PSO, and SA) based SVR forecasting models and three well-known models (RBFNN, MLP-LM, and ARIMA) were selected to compare the forecasting performance. Computational results show that the proposed FA-MA could effectively improve the forecasting accuracy of SVR compared with some other evolutionary algorithms based SVR. Meanwhile, the FA-MA based SVR forecasting model could outperform the selected counterparts significantly. In the second case, comparison results show that the proposed FA-MA is superior to the existing hybrid algorithm in the literature.

However, in this study, only the historical load values are taken into consideration to forecast the electricity load, and some exogenous variables (i.e., temperature, humidity) are also very important to improve the forecasting accuracy. Other topics include more extensive comparison with other models, developing more efficient memetic algorithms and seasonal adjustment. Extensive experimental studies in other forecasting problems and benchmark functions can be investigated. Future work will be on the research of the above cases.

## Figures and Tables

**Algorithm 1:**
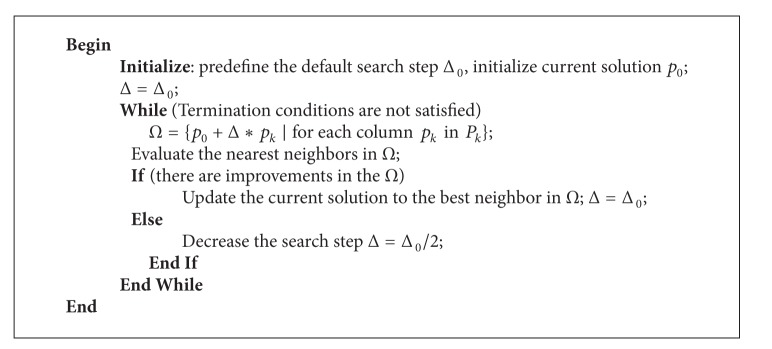
Pseudocode of pattern search for individual learning.

**Algorithm 2:**
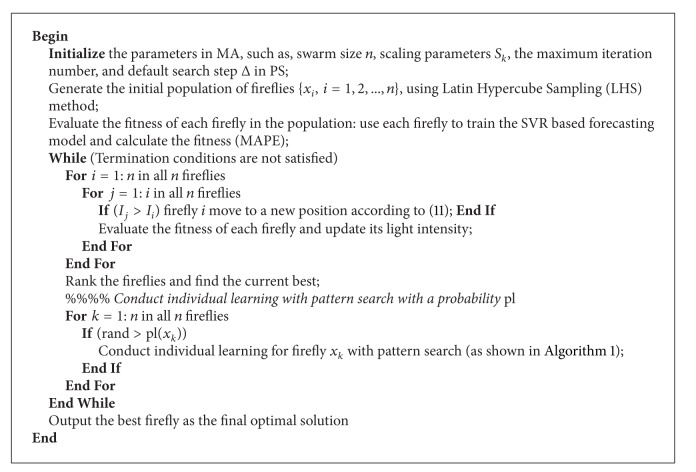
Pseudocode of proposed firefly algorithm based memetic algorithm.

**Table 1:** Performance metrics and their formulas in regression problems.

Metrics	Formula
MAPE	$\begin{array}{c} \;\text{MAPE}=\frac{1}{N}\overset{N}{\underset{i=1}{\sum }}\left|\frac{y_{t+i}-{\hat{y}}_{t+i}}{y_{t+i}}\right|\times 100 \end{array}$
MASE	$\text{MASE}=\frac{1}{N}\overset{N}{\underset{i=1}{\sum }}\left|\frac{y_{t+i}-{\hat{y}}_{t+i}}{\left(1/\left(t-1\right)\right)\sum_{j=2}^{t}\left|y_{j}-y_{j-1}\right|}\right|$
DS	$\text{DS}=\frac{\sum_{i=2}^{N}d_{i}}{N-1}\times 100$ $d_{i}=\{\begin{array}{cc} 1, & \text{if }\left(y_{t+i}-y_{t+i-1}\right)\left(y_{t+i}-{\hat{y}}_{t+i-1}\right)\geq 0\\ 0, & \text{otherwise} \end{array}$

*N* is the number of forecasting periods, *y*_*t*+*i*_ is the actual value at period *t* + *i*, ${\hat{y}}_{t+i}$ is the forecasting value at period *t* + *i*, and $\overset{-}{y}$ is the mean of all values. In this study, the day-ahead (24 hours) short-term load is forecasted recursively, so the number of forecasting periods *N* equals 24.

**Table 2:** Training, validation, and testing set for the first sample case.

Data sets	Period	No. of observation
Training set	1/1/2010–12/31/2010	365∗24
Validation set	1/1/2011–3/31/2011	90∗24
Testing set	4/1/2011–6/30/2011	91∗24

**Table 3:** MAPE (%) of SVR model with different parameter determination methods.

Period	FA-MA	FA	GA	PSO	SA
April	1.24	1.51	1.67	1.73	1.95
May	1.34	1.53	1.77	1.83	2.00
June	1.48	1.78	1.71	2.00	2.14
ALL	1.35	1.61	1.72	1.85	2.03

**Table 4:** MASE of SVR model with different parameter determination methods.

Period	FA-MA	FA	GA	PSO	SA
April	0.33	0.38	0.44	0.47	0.51
May	0.37	0.43	0.50	0.53	0.51
June	0.41	0.48	0.48	0.50	0.56
ALL	0.37	0.42	0.47	0.50	0.53

**Table 5:** DS (%) of SVR model with different parameter determination methods.

Period	FA-MA	FA	GA	PSO	SA
April	96.49	94.74	93.78	93.15	92.77
May	95.73	94.32	93.69	93.08	92.54
June	95.30	93.30	93.50	92.56	91.50
ALL	95.84	94.12	93.66	92.93	92.27

**Table 6:** Time consuming of SVR model with different parameter determination methods.

	FA-MA	FA	GA	PSO	SA
CPU time (min)	27.3	20.7	25.6	21.9	22.5

**Table 7:** MAPE of four forecasting models.

Period	FA-MA	RBF	MLP-LM	ARIMA
April	1.24	2.37	2.35	4.91
May	1.34	2.38	2.39	5.00
June	1.48	2.45	2.51	5.20
ALL	1.35	2.40	2.42	5.04

**Table 8:** MASE of four forecasting models.

Period	FA-MA	RBF	MLP-LM	ARIMA
April	0.33	0.60	0.59	0.72
May	0.37	0.60	0.63	0.75
June	0.41	0.64	0.66	0.78
ALL	0.37	0.61	0.63	0.75

**Table 9:** DS of four forecasting models.

Period	FA-MA	RBF	MLP-LM	ARIMA
April	96.49	90.12	90.34	85.51
May	95.73	89.15	89.01	85.31
June	95.30	89.78	89.19	84.40
ALL	95.84	89.68	89.51	85.07

**Table 10:** Comparison with existing hybrid algorithms.

Period	Actual	TF-*ε*-SVR-SA	CGASA	S-CGASA	FA-MA	S-FA-MA
Oct.08	181.07	184.5035	177.3	175.6385	175.9047	178.2513
Nov.08	180.56	190.3608	177.4428	185.21	184.5484	184.2637
Dec.08	189.03	202.9795	177.5848	189.907	195.4447	188.9679
Jan.09	182.07	195.7532	177.7263	181.9693	185.5828	181.7957
Feb.09	167.35	167.5795	177.8673	163.2805	161.4537	161.9352
Mar.09	189.3	185.9358	178.0078	182.1747	184.854	181.9227
Apr.09	175.84	180.1648	178.6806	177.6289	177.2037	176.1128

MAPE (%)		3.799	3.731	1.901	2.433	1.583
MASE		0.576	0.554	0.237	0.326	0.217
DA (%)		83.333	33.333	83.333	83.333	83.333
